# Damage Evolution and Lifetime Prediction of Cement Asphalt Mortar Under High-Speed Train Frequency and Temperature Gradient Load

**DOI:** 10.3390/ma18051011

**Published:** 2025-02-25

**Authors:** Mingjie Zhou, Shenghua Zhong, Yiping Liu, Zejia Liu, Bao Yang, Zhenyu Jiang, Licheng Zhou, Liqun Tang

**Affiliations:** School of Civil Engineering and Transportation, South China University of Technology, Guangzhou 510640, China; 202220107159@mail.scut.edu.cn (M.Z.); z15170289417@163.com (S.Z.); byang20210415@scut.edu.cn (B.Y.); zhenyujiang@scut.edu.cn (Z.J.); ctlczhou@scut.edu.cn (L.Z.); lqtang@scut.edu.cn (L.T.)

**Keywords:** cement asphalt mortar (CA mortar), high-speed train, temperature gradient load, damage evolution, lifetime prediction

## Abstract

Severe damage to cement asphalt mortar (CA mortar) can compromise the stability and safety of high-speed railway operations due to various complex factors during service. The loads from high-speed trains and temperature gradients within the ballastless track structure are significant contributors to this damage. However, most previous studies have focused on laboratory tests or numerical simulations under simple loading conditions, while few have investigated the damage evolution of CA mortar when both train loads and temperature gradients are considered simultaneously. In this paper, a finite element model of the CRTS II ballast track and a high-speed railway train dynamics model based on the damage constitutive model of CA mortar was established. The damage evolution of CA mortar through long-term cyclic numerical simulations under the combined effects of train load and temperature gradient load were investigated. By integrating the maintenance criteria for high-speed railways, the lifetime of CA mortar using the criteria of crack length and off-seam width was predicted. In addition, the material and structural properties of CA mortar were also optimized, considering the relationship between its elastic modulus and density, to enhance its lifetime. The conclusions reached are more realistic. The results indicate that the combined load causes deformation in the ballast track structure, leading to gradual damage progression from the edge to the interior of the CA mortar layer. The lifetime of CA mortar is determined by the number of days it takes for the crack length to reach the maintenance criteria. The lifetime of CA mortar under different temperature gradients ranges from 1 to 2 years. Increasing the elastic modulus and thickness of the CA mortar layer improves its lifespan. An elastic modulus of 9000 MPa and a thickness of 50 mm for the CA mortar were recommended.

## 1. Introduction

High-speed railways have become a vital component of modern transportation infrastructure, with China’s network exceeding 40,000 km in length and serving billions of passengers annually. It is important to ensure the comfort and safety of high-speed trains during operation, where the track structure must have the characteristics of high smoothness and high stability. Current track structures can be divided into ballasted and ballastless tracks. It has been proven that a ballastless track allows the train to run more smoothly and at a higher speed. For this reason, most high-speed railways are built with ballastless tracks.

High-speed railway ballastless track structures are primarily categorized into CRTS I, II, and III type plate ballastless tracks and CRTS I and II type double-block ballastless tracks. Among these, the plate ballastless track structure is notable for its excellent structural stability, rigidity, smoothness, and durability and has been widely used in high-speed railways [1]. The plate ballastless track system mainly consists of rails, fastening systems, track plates, CA mortar filling layers, and base plates. Among these components, cement asphalt mortar (CA mortar) is the primary constituent of the filling layer. CA mortar possesses excellent dynamic [2,3] and damping [4] properties, playing a crucial role in the track structure by providing support, load transfer, and vibration isolation. Its performance directly affects the safety of the track structure and the comfort of train operations. CA mortar is considered the core technology of slab tracks. The performance of CA mortar is highly sensitive to environmental factors, and its dynamic and damping properties are crucial for maintaining track stability. However, during operation, CA mortar is subjected to complex loads and harsh climatic conditions. Common damages include edge cracking, block fracture, and off-seam from the track plate. These damages lead to a deterioration in its mechanical properties, seriously affecting the smoothness, safety, and comfort of high-speed rail operations. Due to the lack of effective repair technologies for the filling layer, the only option for damaged CA mortar layers is to replace them entirely. This process is not only complicated and expensive but also significantly disrupts railway operations [5]. Damage to the CA mortar layer has become one of the most challenging issues in railway system operations.

One of the main causes of severe CA mortar damage is the complex and long-term loads it experiences. CA mortar is subjected to fatigue loads due to the continuous passage of high-speed trains over extended periods. Additionally, because the mechanical properties of CA mortar are highly sensitive to temperature [6,7], it is also significantly affected by the varying temperature gradient loads within the track structure. The mechanical response of CA mortar under the combined effects of train fatigue loads and temperature gradient loads can reflect its service conditions in the railway system. Therefore, studying the damage evolution of CA mortar under the combined loading of train loads and temperature gradient loads and predicting its lifespan is of great research significance in addressing the serious problem of CA mortar damage.

Some scholars have conducted research on enhancing the cement product’s strength and durability. Wang et al. [8] developed a stress–strain model to describe the performance of CA mortar at low temperatures. Liu and Wang et al. [9,10] studied the compressive strength of CA mortar under various temperature conditions. Fu et al. [6] investigated the temperature sensitivity of CA mortar and established a model for stress relaxation properties. Wang et al. [11] developed a stress–strain model for CA mortar subjected to both temperature and loading rate. Wang et al. [12] investigated the fatigue behavior when CA mortar is exposed to water. Wang et al. [13] studied the fatigue life of combined structures of different hot-mix asphalt and CA mortar. Ren et al. [14] study criteria for repairing damages of CA mortar. Zhao et al. [15] conducted fatigue tests under coupled loading conditions. Hosseinzadehfard et al. [16] studied concrete mixed with both micro silica and natural zeolite to enhance the strength and durability of concrete. Wang et al. [17] studied the working properties, mechanical properties, and freezing resistance of CA mortar. Zhu et al. [18] explored the mechanisms by which high temperatures influence CA mortar creep based on several microscopic testing methods.

The mechanical behavior of concrete structures has also been explored. Chen et al. [19] examined the influence of CA mortar’s elastic modulus on the stress and deformation of slab tracks under train loads and temperature gradients. Peng et al. [20] found that the interfacial bonding strength between CA mortar and track slabs is affected by temperature. Zeng et al. [21] studied the deterioration of CA mortar layers under cyclic thermal loads. Fu et al. [22] analyzed the mechanical response of CA mortar based on temperature fields. Fu and Zhou et al. [23] investigated the deformation properties of CA mortar under different temperatures. Wang et al. [24] discovered that the degradation of CA mortar is primarily caused by increased stress and the deterioration of tensile capacity due to extreme climates. Yao et al. [25] studied the interface performance of CA mortar under temperature loads. Li et al. [26] analyzed off-seam damage under both temperature and train loads using the finite element method. Deng and Du et al. [27,28] studied the fatigue damage evolution of CA mortar by establishing the finite element model. Qiu et al. [29] investigated the coupling effect of temperature and fatigue loading on CA mortar. Shan et al. [30] studied the fatigue performance of CA mortar under simulated service conditions. Zhou et al. [31] investigated the thermal response of the bridge-supported CRTS II slab track system through experimental testing. Liu et al. [32] analyzed the distribution and change rules of the temperature field of CRTS II slab ballastless track. Mobaraki et al. [33] used a comprehensive numerical approach to study the impact of a protective barrier on the response of a box-shaped tunnel. Feng et al. [34] proposed a method for analyzing the reliability of the ballastless track system affected by a CA mortar gap during service life. Chen et al. [35] developed a new coupled thermal-mechanical model for the concrete slab track at the mesoscale to predict the life of CA mortar.

Summarizing the current research on the damage characteristics of CA mortar under temperature gradient and train loads, several deficiencies are evident: most studies do not consider the scheduled departures of high-speed trains when applying train fatigue loads; few studies account for cycling temperatures when applying temperature gradient loads; and there is a relative lack of research on the combined effects of temperature gradient and train loads. This paper establishes a non-linear CA mortar damage constitutive model to calculate its plasticity parameters and uses ABAQUS 2021 to create a finite element model. A high-speed railway train dynamics model is developed in the multi-body dynamics analysis software SIMPACK 2021 to obtain wheel-rail forces during high-speed train travel. This study applies train loads and temperature gradient loads to the finite element model and uses numerical simulation results to investigate the damage evolution of CA mortar. The lifetime of CA mortar is predicted based on maintenance criteria from the Chinese ballastless track structure specifications, and the properties of CA mortar are optimized based on their lifetime.

This study distinguishes itself from prior research by rigorously addressing the coupled effects of high-speed train frequency and temperature gradient loads on CA mortar degradation. While existing works predominantly focus on isolated mechanical or thermal loading conditions, the synergy between dynamic train-induced stresses and cyclic thermal expansion–contraction remains poorly quantified. Such coupled interactions are vital because temperature fluctuations modulate CA mortar’s viscoelastic behavior, and repeated train passages may intensify it. By integrating a damage-constitutive finite element model with train dynamics simulations, this work pioneers a framework for predicting long-term CA mortar performance under realistic service conditions. The proposed methodology not only advances the mechanistic understanding of CA mortar but also addresses an urgent industry demand for maintenance strategies, particularly in regions experiencing extreme climatic variations.

## 2. Damage Constitutive Model and Numerical Simulation Model

### 2.1. Damage Constitutive Model

In this study, the Concrete Damaged Plasticity (CDP) model was employed to characterize the damage in CA mortar. The CDP model is a continuum-based plastic damage model designed for analyzing materials with concrete-like properties. It posits that concrete primarily exhibits two damage modes: tensile cracking and compressive crushing. The evolution of its yield surface is governed by two hardening parameters, which correspond to the damage modes under tensile and compressive loading, respectively. The model primarily requires the user to specify the plastic mechanical behavior of the material under both uniaxial tension and compression.

Since the CDP model is driven by inelastic strains, it is necessary to establish the relationships between tensile stress and inelastic strain (*σ_t_*—*ε_ck_*), tensile damage variable and inelastic strain (*D_t_*—*ε_ck_*), compressive stress and inelastic strain (*σ_c_*—*ε_in_*), and compressive damage variable and inelastic strain (*D_c_*—*ε_in_*). All these parameters are derived from the damage constitutive model. To obtain the plastic damage parameters for CA mortar, the stress–strain relationship curves under both tensile and compressive conditions must be determined.

Based on the “parallel rod model”, it is assumed that the components of CA mortar consist of a system of *N* microscopic rod elements arranged in parallel. These microscopic rods behave as linear elastic bodies before damage occurs but lose their load-bearing capacity after damage. The viscous component within the CA mortar does not sustain damage and deforms in coordination with the microscopic rods. The strain of each microscopic rod that undergoes damage follows the Weibull distribution. Based on the established experimental and theoretical studies on the properties of CA mortar [36], the following equation was chosen to describe the stress–strain relationship of CA mortar:

(1) $$\sigma =E\varepsilon \exp\left[-{\left(\frac{\varepsilon }{m}\right)}^{n}\right]+\eta \theta$$

where *ε* is the strain, *D* is the damage variable, *η* is the viscosity coefficient of CA mortar, *θ* is the strain rate, and *m* and *n* are the distribution parameters of the Weibull distribution.

However, the product of the viscosity coefficient and strain rate has a negligible effect compared to its peak compressive strength. According to the commonly used concrete-like material damage principal relationship equation, *σ = E*(1 − *D*)*ε*, the following relationship between the damage variable *D* and the strain *ε* is derived:

(2) $$D=1-\exp\left[-{\left(\frac{\varepsilon }{m}\right)}^{n}\right]$$

From the known stress–strain test curves of CA mortar, it can be observed that at the peak point of the curve (*σ_max_*, *ε_max_*), the derivative dσ/dε = 0 is given by the following:

(3) $$E\exp\left[-{\left(\frac{\varepsilon_{\max}}{m}\right)}^{n}\right]\left[1-n{\left(\frac{\varepsilon_{\max}}{m}\right)}^{n}\right]=0$$

Obviously, the first and second terms on the left side of the equation are not equal to zero. Therefore, the following occurs:

(4) $$\left[1-n{\left(\frac{\varepsilon_{\max}}{m}\right)}^{n}\right]=0$$

We can obtain the following:

(5) $$m=\frac{\varepsilon_{\max}}{{\left(\frac{1}{n}\right)}^{\frac{1}{n}}}$$

When σ = σ_max_ and ε = ε_max_, by substituting Equation (6) into Equation (1), we obtain the following:

(6) $$n=\frac{1}{\ln\frac{E\varepsilon_{\max}}{\sigma_{\max}}}$$

**Figure 1:**
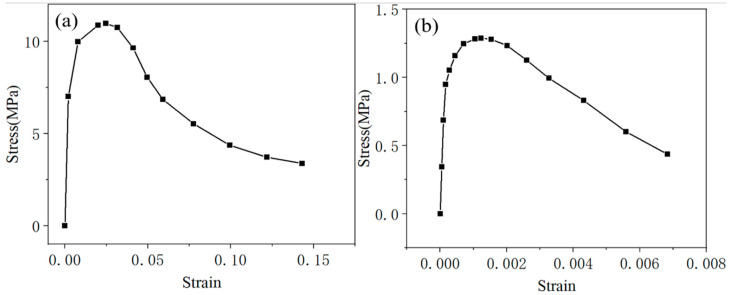
Stress–strain curve for CA mortar: (**a**) compressive stress–strain relationship, (**b**) tensile stress–strain relationship.

**Figure 2:**
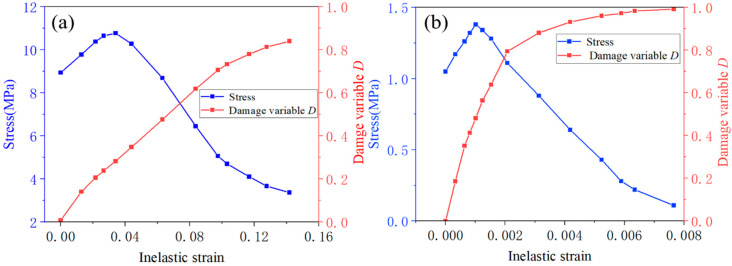
Stress—inelastic strain and damage variable—inelastic strain curve for CA mortar: (**a**) compressive properties and (**b**) tensile properties.

The compressive and tensile stress–strain relationships of CA mortar were derived from the uniaxial compressive test reported in the literature [37] and the splitting tensile strength test described in the literature [38], as illustrated in Figure 1. After calculating the distribution parameters *m* and *n* from Equations (6) and (7) using the stress–strain curve, the relationship between the damage variable *D* and the strain *ε* can be determined from Equation (4). Additionally, the relationship between strain and inelastic strain can be established based on the definition of inelastic strain. With these results, the relationships between tensile stress and inelastic strain (*σ_t_*—*ε_ck_*), tensile damage factor and inelastic strain (*D_t_*—*ε_ck_*), compressive stress and inelastic strain (*σ_c_*—*ε_in_*), and compressive damage factor and inelastic strain (*D_c_*—*ε_in_*) can be obtained, as shown in Figure 2. All these parameters will be input into ABAQUS to establish the finite element model of the ballastless track.

### 2.2. Finite Element Model of CRTS II Ballastless Track

In the numerical simulation, the elastic modulus, density, and thickness of the CA mortar layer are the primary physical properties that are controlled. According to the specifications for CA mortar, the elastic modulus should be maintained within the range of 7000 MPa to 10,000 MPa, and its density should be no less than 1700 kg/m^3^. Furthermore, in accordance with the specifications for ballast track structures, the thickness of the CA mortar layer should be controlled between 30 mm and 60 mm. Controlling these properties is essential to investigate the trend of damage evolution in CA mortar as its physical characteristics vary.

**Figure 3:**
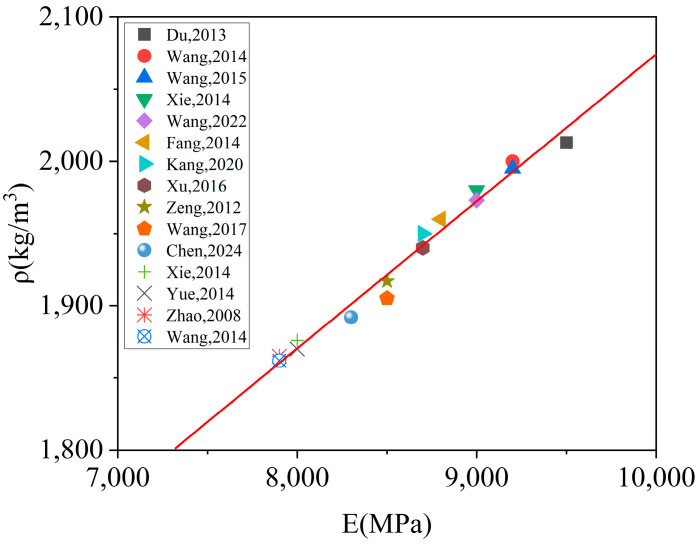
Density and elastic modulus of various CA mortars [17,35,39,40,41,42,43,44,45,46,47,48,49,50,51].

For materials similar to concrete, there is typically a positive correlation between elastic modulus and density. To explore this relationship, data from fifteen literature [17,35,39,40,41,42,43,44,45,46,47,48,49,50,51] that conducted tests on CA mortar were selected. The elastic modulus and density of the prepared CA mortar samples, as reported in the literature, were plotted as scatter plots in Figure 3. Analysis of the scatter distribution in Figure 3 revealed a positive linear correlation between the elastic modulus and density of CA mortar. A linear fit was applied to the scatter points in Figure 3, and the resulting linear equation is presented in Equation (7). Here, the unit of density *ρ* is kg/m^3^ and the unit of elastic modulus *E* is MPa. With this established relationship between the density and elastic modulus for CA mortar, adjusting the elastic modulus effectively allows for simultaneous control over the density.

(7) ρ=0.10E+1057.44

**Figure 4:**
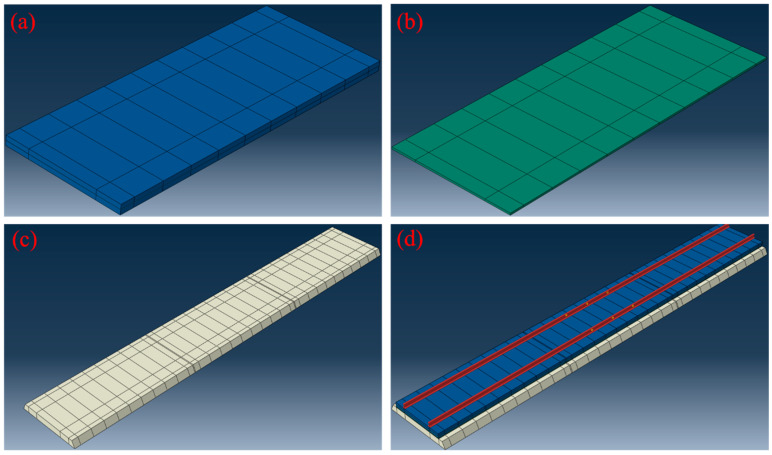
Finite element model of CRTS II ballastless track: (**a**) track plate, (**b**) CA mortar layer, (**c**) base plate, and (**d**) overall structure.

Based on Equation (7) and the specifications for ballast tracks in China, the properties required for the model calculations used in this paper are summarized in Table 1. Here, *ρ* represents the density, *E* denotes the modulus of elasticity, *λ* is Poisson’s ratio, *α* is the coefficient of thermal expansion, *k_x_* and *k_z_* represent the transverse and vertical stiffness of rail fasteners, respectively, and *k_y_* denotes the longitudinal resistance. The finite element model of the CRTS II type plate ballastless track structure is depicted in Figure 4. The CDP model was employed to characterize the damage behavior of CA mortar. The stress–inelastic strain and damage factor–inelastic strain relationship under tension and compression were derived from Figure 2. The CA mortar contained no initial cracks or debonding defects. A finite element model was constructed, incorporating three track plates along with their corresponding CA mortar layers beneath. The base plate was modeled as a continuous single piece positioned underneath the CA mortar layers. The model’s boundaries were fully constrained at both ends, with expansion joints introduced between each track plate and the CA mortar layer. Additionally, the bottom of the support layer was connected to a foundation characterized by specific stiffness and damping properties. Since the model includes three pieces of track slab and the CA mortar layer, the results from the middle section will be utilized for numerical simulation to mitigate boundary effects.

**Figure 5:**
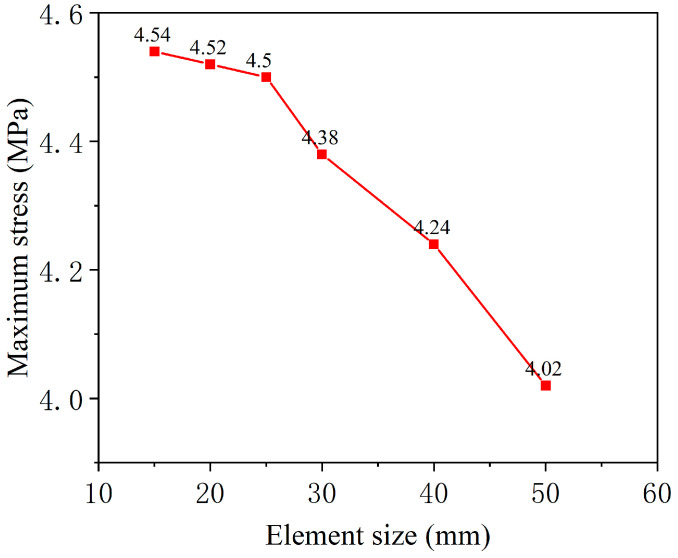
Maximum stress values for different element sizes.

To determine the appropriate mesh size for the finite element model, a mesh sensitivity analysis was conducted. Six different element grid lengths were compared: 50 mm, 40 mm, 30 mm, 25 mm, 20 mm, and 15 mm. A temperature gradient load of 60 °C/m was applied to the model, and the computational results for each case were analyzed. Figure 5 illustrates the maximum stress values in the CA mortar layer for different element sizes. As the element size gradually decreased, the stress values increased correspondingly. However, the stress variation stabilized when the element grid length was ≤25 mm. Specifically, the maximum stress for the 15 mm element size was only 0.89% higher than that for the 25 mm, whereas the maximum stress for the 25 mm element size was 8.95% higher than that for the 50 mm. Reducing the number of elements appropriately is beneficial for improving computational performance. Therefore, the element grid length for the CA mortar layer in the model was set to 25 mm. The finite element model has a total of about 1.5 × 10^6^ elements.

### 2.3. High-Speed Railway Train Dynamics Model

**Figure 6:**
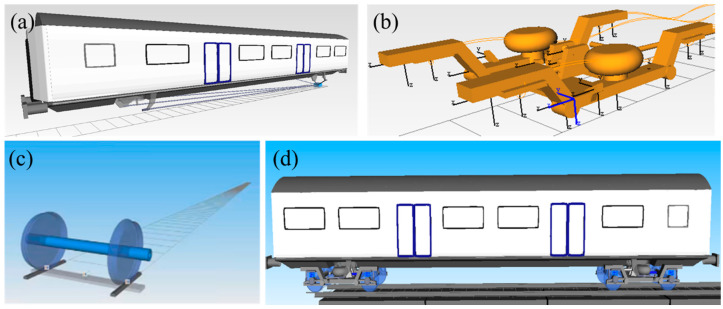
High-speed train mode: (**a**) vehicle body, (**b**) bogie, (**c**) wheel, and (**d**) whole model.

In this section, a high-speed railway train dynamics model is developed using SIMPACK, based on a theoretical system of vehicle–track coupling dynamics. The dynamic model is set up on the basis of the Chinese Fuxing CR400AF high-speed railway train, and the specific structural parameters of this train are shown in Table 2. The vehicle model is constructed as a multi-rigid body coupled motion system comprising the vehicle body, bogies, wheel pairs, and suspension springs. The vehicle body is modeled with 5 degrees of freedom, including nodding, floating, traversing, side-rolling, and head-shaking motions. The bogies are assigned 10 degrees of freedom, encompassing nodding, floating, traversing, side-rolling, and head-shaking motions. The wheels are allocated 16 degrees of freedom, which include sinking, floating, traversing, side-rolling, and head-shaking motions. In total, the vehicle model has 31 degrees of freedom. The resulting high-speed train model is illustrated in Figure 6.

### 2.4. Model Reliability Verification

In the literature [27], the ballastless track on the Sui-Yu line in China has been dynamically tested, and some test data has been obtained, which is used in this section to compare with the numerical simulation results. In the simulation, both train loads and temperature gradient loads are applied to the ballasted track. As the maximum speed of the high-speed train on the line is 300 km/h, the data when the train passes at the speed of 300 km/h are used for comparison. The results of the comparison are shown in Table 3. The simulation results differ from the test results due to some differences between the simulation model and the actual situation, but the difference is within a reasonable range, so the model developed in this paper is reliable.

## 3. Damage Evolution and Lifetime Prediction of CA Mortar Layer

Each component of the ballastless track has its own maintenance criteria. The CA mortar layer, in particular, is maintained on an annual cycle, with two primary maintenance criteria: crack length and off-seam width from the track slab. These criteria correspond to the two forms of damage: cracking and off-seaming, respectively. When the crack length is between 20 and 50 mm, or the off-seam from the track plate is between 1 and 1.5 mm, the maintenance criteria trigger Level A “Attention required”. For crack lengths between 50 and 100 mm or off-seams between 1.5 and 2 mm, Level B “Scheduled repair” is triggered. If the crack length exceeds 100 mm or the off-seam is greater than 2 mm, Level C “Immediate repair” is triggered [52]. During maintenance, appropriate measures are taken based on the different levels of damage. Crack lengths and off-seam widths are the easiest to detect and quantify. Other modes of damage, such as internal delamination and interfacial bond failure, are ultimately reflected in the formation of cracks and off-seam. This criterion synthesizes the standards and regulations from various countries and is applicable to the construction of high-speed railways worldwide. In the subsequent analyses in this paper, the criteria will be used as a reference. When the condition of the CA mortar reaches the “Immediate repair” level, it is considered that the damage to the CA mortar is so severe that it requires maintenance and has reached the end of its service life.

**Figure 7:**
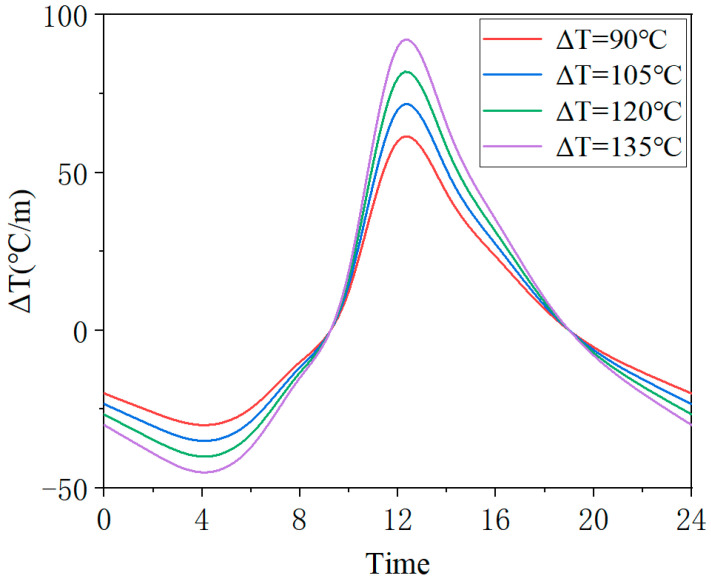
Variation of temperature gradient loads over time.

The investigation of CA mortar damage evolution necessitates long-term cyclic numerical simulations, with this study employing a daily cycle as the fundamental unit. Two types of loads—temperature gradient load and train load—are applied to the model within each day. The temperature gradient of the ballast track varies continuously throughout the day. Since different external temperature environments also influence the temperature gradient loads within the ballast track, it is essential to compare and analyze the damage evolution of the CA mortar layer under various temperature gradient loads. Table 4 presents the maximum positive and negative temperature gradients for four different temperature gradient loads used in this study’s numerical simulation, where ΔT represents the difference between the maximum positive and negative temperature gradients. According to the standard [52], the temperature gradient corresponding to Condition No. 1 represents the extreme operational temperature gradient. Based on the tested temperature gradient values over different days in reference [31], the maximum positive temperature gradient within a day reaches at least 60 °C/m, while the maximum negative temperature gradient within a day reaches at least 30 °C/m. Therefore, these values were adopted as the temperature gradient for serial number 4. For ΔT, a step size of 15 °C/m was used to determine the maximum temperature gradient values corresponding to Condition No. 2 and 3. The maximum temperature gradients typically occur at 12 a.m. and 4 a.m. The temporal variation of these four temperature gradient loads is depicted in Figure 7, with the loads applied to the ballastless model according to the temperature gradient changes at different times.

A passing high-speed railway train exerts multiple vehicle loads on the ballast track. Based on the structure of the CR 400 AF high-speed train, each train consists of 8 carriages, with each carriage having 2 bogies and 4 pairs of wheels. Consequently, each passing train generates 8 × 2 × 4 = 64 vehicle loads on the ballastless track. Table 5 illustrates the schedule on the Guangzhou-Shenzhen railway at various times on a given day, serving as a reference for the train loads applied within a day. In the simulation, the frequency of train loads applied corresponds to the number of train departures at different times. From 0 a.m. to 6 a.m., no trains depart due to maintenance, and during this period, the ballastless track is solely affected by temperature loads.

### 3.1. CA Mortar Cracked Damage

**Figure 8:**
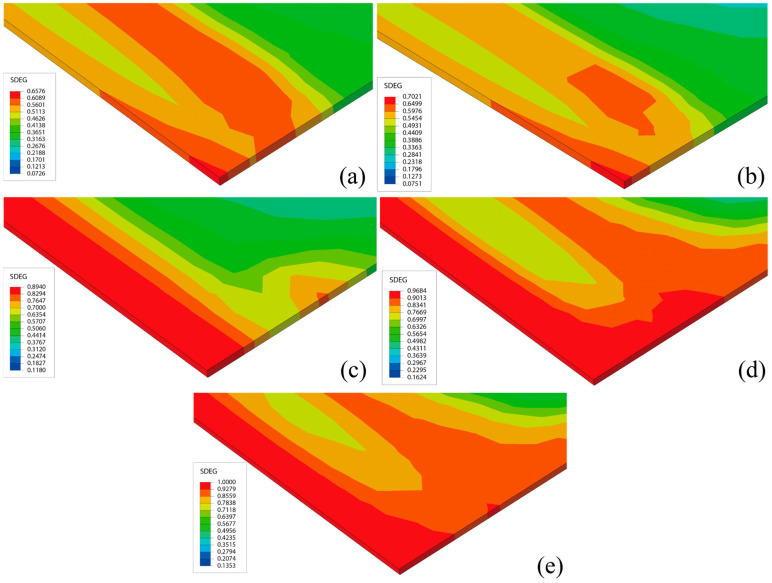
Distribution of damage variables over different days: (**a**) day 240: *D_max_* = 0.6575, (**b**) day 270: *D_max_* = 0.7021, (**c**) day 360: *D_max_* = 0.8940, (**d**) day 450: *D_max_* = 0.9684, (**e**) day 566: *D_max_* = 1.

In the numerical simulation, the element grid length of the CA mortar is set to 25 mm. When the damage variable of four consecutive adjacent elements in the same direction reaches 1, this indicates that the crack length has reached 100 mm, thus meeting the “Immediate repair” level. At this point, the crack damage in the CA mortar is considered severe enough to render it unusable in service. Figure 8 illustrates the distribution of damage variables over different days when ΔT = 90 °C. According to this distribution, it is evident that the regions with severe damage are located at the edges and corners of the layer. The stress concentration resulting from the overall deformation of the ballast track significantly accelerates the development of damage in the CA mortar layer.

**Figure 9:**
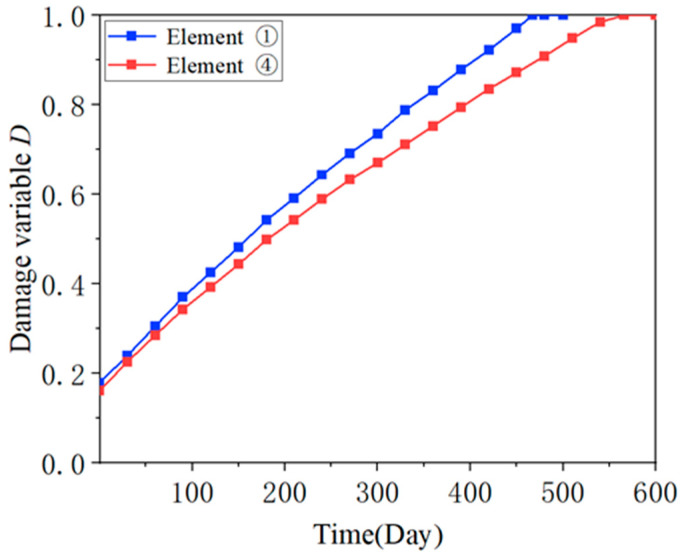
Evolution of damage variables.

In the simulation, the damage variables of four consecutive adjacent elements at the corners of the slab along the transverse direction are consistently larger. To differentiate them, these four consecutive adjacent units are numbered from the outside to the inside as ① to ④. When the simulation reached day 467, the damage variable of unit ① reached 1, and macroscopic damage began to appear in the CA mortar layer. However, at this time, the cracked area in the CA mortar layer was relatively small and had not yet met the “immediate repair” level according to the maintenance standards. On day 566, the damage variable of unit ④ also reached 1. At this point, all four consecutive adjacent units had damage variables of 1, and the CA mortar layer reached the “ immediate repair” level. Figure 9 shows the evolution of the damage variables of unit ① and unit ④ over the course of the simulation: initially, each element had a small initial damage, which gradually increased to 1 as the number of days increased, with a smooth growth rate and minimal change.

**Figure 10:**
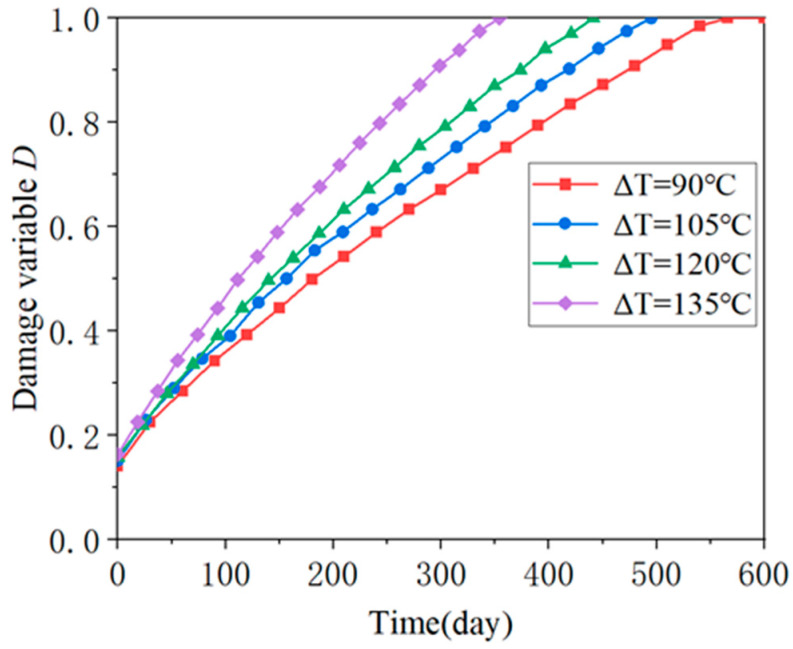
Evolution of damage variable of unit ④ under different temperature gradient loads.

**Figure 11:**
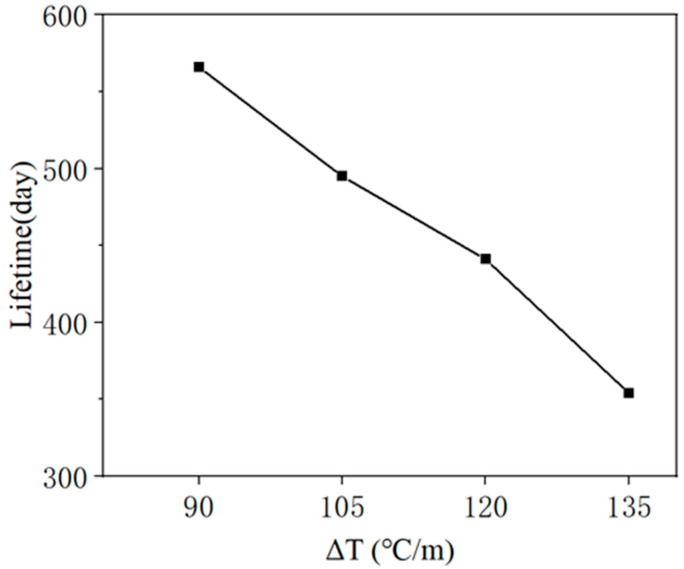
Relationship between lifetime and ΔT.

Figure 10 illustrates the evolution of the damage variable of unit ④ under different temperature gradient loads, which can indicate when the CA mortar layer reaches the “immediate repair” level under varying temperature gradients. Figure 11 shows the relationship between lifetime and ΔT. As ΔT increases, there is a clear trend of more rapid deterioration in the CA mortar layer. In the extreme case where the ballastless track is subjected to a temperature gradient of ΔT = 135 °C/m, the CA mortar enters the “immediate repair” level after 354 days. In a milder situation with ΔT = 90 °C/m, it takes 566 days for the CA mortar to reach this level. Therefore, according to the simulation, most CA mortar layers can serve in ballastless tracks for approximately 1 to 2 years. This aligns with the 1-year maintenance cycle mentioned in previous studies, reflecting the reasonableness of the maintenance specifications for high-speed railways.

### 3.2. CA Mortar Off-Seam Damage

Off-seam separation of the CA mortar layer from the track slab is another major form of damage in addition to cracking. When subjected to complex loads from the external environment, the bond between the CA mortar layer and the track slab may no longer be able to withstand the loads, leading to the separation of the upper and lower layers. In the numerical simulation, friction between the track slab and the CA mortar layer ensures that their displacements remain synchronized until the load exceeds the threshold; beyond this point, the track slab separates from the CA mortar layer, and their displacements diverge. In this section, the off-seam width is defined as the difference in vertical displacement between the track slab and the CA mortar layer. Since the CA mortar reached the “immediate repair” level on day 566 in the mildest case (ΔT = 90 °C) during the crack damage analysis, all off-seam numerical simulations were conducted for 566 days.

**Figure 12:**
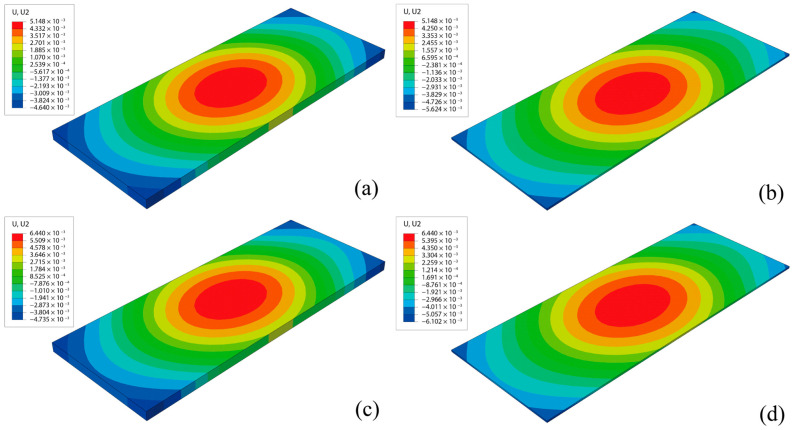
Vertical displacement of track slab and CA mortar: (**a**) track slab, Day 480; (**b**) CA mortar layer, Day 480; (**c**) track slab, Day 566; (**d**) CA mortar layer, Day 566.

Figure 12 displays the vertical displacement of the track slab and CA mortar at certain simulation days under the condition of ΔT = 90 °C. The comparison of vertical displacement cloud plots reveals minimal differences between the track slab and the CA mortar layer near the central region of the slab. However, the vertical displacement difference gradually increases from the central region towards the edge and peaks at the corners, where the most severe crack damage occurs. The stress concentration at the corners, due to the deformation of the ballastless track, exacerbates both types of damage in this area.

**Figure 13:**
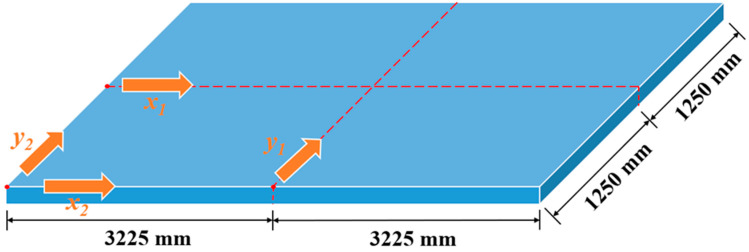
Schematic diagram of CA mortar layer.

**Figure 14:**
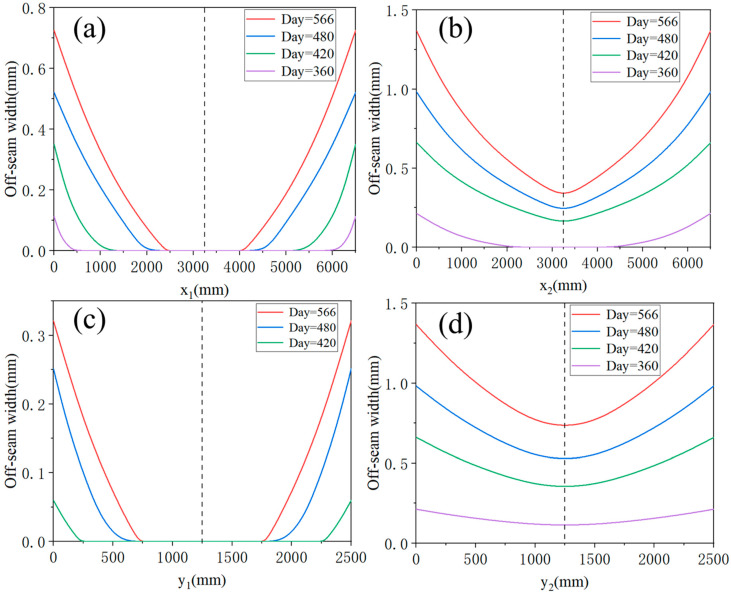
Distribution of off-seam width in four directions: (**a**) *x*_1_, (**b**) *x*_2_, (**c**) *y*_1_, (**d**) *y*_2_.

The distribution of off-seam width can also be analyzed. Figure 13 presents a schematic diagram of the CA mortar layer, marking four directions: direction *x*_1_—the center of the slab along the longitudinal direction; direction *x*_2_—the edge of the slab along the longitudinal direction; direction *y*_1_—the center of the slab along the transverse direction; direction *y*_2_—the edge of the slab along the transverse direction. Figure 14 illustrates the distribution of off-seam width along these four directions on different days. In Figure 14, the vertical axis represents the off-seam width, while the horizontal axis indicates the distance from the starting point in each direction. In the transverse direction, the off-seam width initially decreases and then increases, gradually narrowing from the edge toward the center of the plate and being symmetrically distributed. At the same distance, the off-seam width in direction *y*_2_ is greater than that in direction *y*_1_, and off-seams appear in direction *y*_2_ before they do in direction *y_1_*. After 566 days, some areas in direction *y*_1_ still do not exhibit off-seams. The distribution of off-seams in the longitudinal direction mirrors that in the transverse direction, with the off-seam width also decreasing first and then increasing symmetrically. At the same distance, the off-seam width in direction *x*_2_ is greater than that in direction *x*_1_, and some areas in direction *x*_1_ remain off-seam-free until day 566. The off-seam width is larger in the edge regions (directions *x*_2_ and *y*_2_) compared to the central regions (directions *x*_1_ and *y*_1_), with the maximum off-seam width occurring at the corners of the plate.

**Figure 15:**
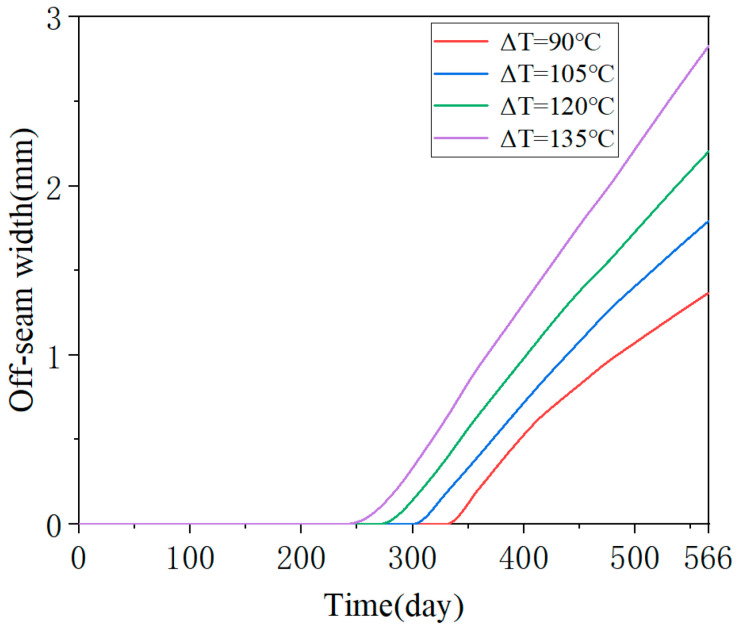
Evolution of maximum off-seam width over days for different working conditions.

**Figure 16:**
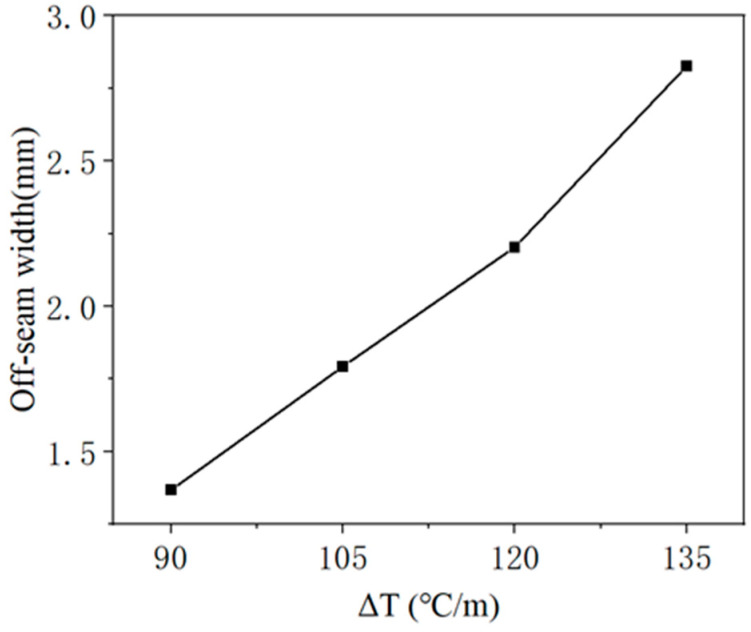
Relationship between off-seam width and ΔT on day 566.

Similar to the analysis of crack damage, the off-seam damage analysis necessitates a comparative study of the damage evolution of the CA mortar layer under various temperature gradient loads. The temperature gradient loads are described in Table 4 and Figure 7. Figure 15 presents the curves of the maximum off-seam width of CA mortar over time under different working conditions. Under mild conditions (ΔT = 90 °C/m), the CA mortar began to exhibit off-seam damage with the track slab on day 316. As ΔT increased, the onset of off-seam damage occurred earlier: it appeared on day 281 for ΔT = 105 °C/m, on day 251 for ΔT = 120 °C/m, and on day 225 for ΔT = 135 °C/m. Figure 16 illustrates the relationship between off-seam width and ΔT on day 566. As ΔT increases, the off-seam width at the end of the calculation also increases. For the different cases, ranging from the most extreme to the mildest conditions, the off-seam widths on day 566 reached 2.826 mm, 2.203 mm, 1.792 mm, and 1.367 mm, respectively. In the two milder cases, the off-seam widths did not exceed the rapid repair criterion of 2 mm. However, in the more extreme cases, the off-seam widths surpassed 2 mm, indicating that repairs were necessary. These results demonstrate that the temperature gradient significantly influences the separation behavior between the CA mortar and the track slab.

### 3.3. Lifetime Prediction

When comparing the damage evolution processes of cracking and off-seaming, it is observed that the off-seam width does not reach 2 mm when the crack length reaches 100 mm in all cases. Therefore, it can be roughly assumed that using the criterion for assessing damage evolution, the rate of crack damage development in the CA mortar layer is slightly faster than that of off-seam, making crack damage more likely to reach the “immediate repair” threshold. However, this analysis is not sufficiently intuitive, necessitating a comparative analysis under different working conditions. According to the specifications, the elastic modulus of CA mortar should be controlled within the range of 7000 MPa to 10,000 MPa [52], and the range of elastic modulus in previous studies (Figure 3). Four types of CA mortar with varying elastic moduli were selected for numerical simulation: 7000 MPa, 7500 MPa, 8000 MPa, and 8500 MPa. The train load was applied in the same manner as before, with ΔT = 90 °C. The calculation was halted when the off-seam width reached 2 mm.

**Figure 17:**
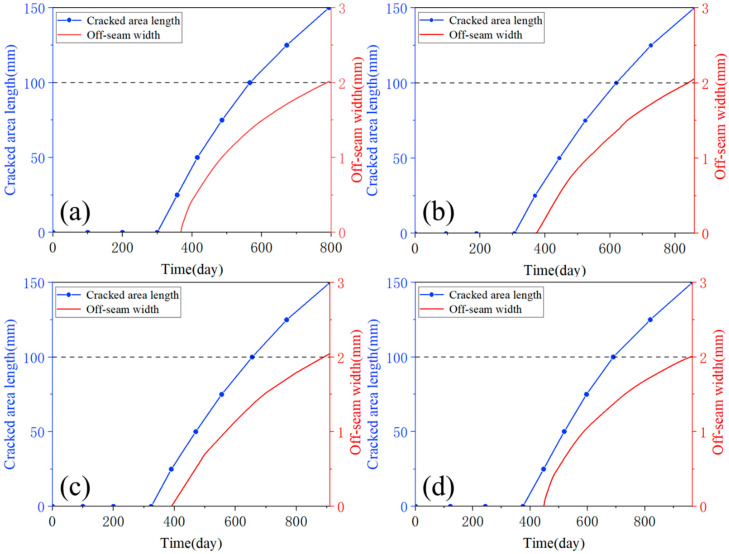
Comparison of crack length and maximum off-seam width: (**a**) E = 7000 MPa, (**b**) E = 7500 MPa, (**c**) E = 8000 MPa, (**d**) E = 8500 MPa.

Figure 17 illustrates the evolution of crack length and maximum off-seam width over time for different conditions. Since the length of each element in the finite element model is set to 25 mm in this study, the development curve of damage length over time can only be extracted when the damaged area length reaches a multiple of 25 mm, with scattered points connected to form the graph. As shown in Figure 17, the development trends of crack length and off-seam width are essentially similar across the four cases, leading to the assumption that the damage evolution trend is similar for all other conditions. So, it can be obtained that the choice of elastic modulus does not actually have a significant effect on this part of the analysis.

From Figure 17, it is evident that the rate of development for both the crack length and the maximum off-seam width slows down over time. In Figure 17, the vertical coordinates of (a) to (d) are consistent: double y-axis plots, with the “immediate repair” criteria of 100 mm for crack damage and 2 mm for off-seam damage aligned at the same height on the double y-axis plots to ensure equal scales. This facilitates a visual comparison of the evolution of both types of damage. The two curves do not intersect, and the development curve of the crack length is consistently above that of the maximum off-seam width. Table 6 delineates the days on which cracked area and off-seams appear under four cases, along with the days on which damaged cracked length and off-seam width reach “immediate repair” criteria, as well as the corresponding intervals required to attain the criteria. The days on which crack damage appears are earlier than those for off-seam damage, and the days on which the “immediate repair” level is reached are also earlier. It also took less time for the cracked area length to reach the criteria than for the off-seam width to reach the criteria. It can be concluded that, according to the maintenance criteria, the rate of crack damage development in the CA mortar layer is slightly faster than that of off-seam damage. Therefore, it can be concluded that the lifetime of CA mortar primarily depends on when the crack length reaches 100 mm. For example, the number of days for the damaged area length to reach 100 mm when ΔT is 90 °C, 105 °C, 120 °C, and 135 °C are 566 days, 495 days, 441 days, and 354 days, respectively, with the lifetime of the CA mortar corresponding to these respective days.

## 4. Optimization of CA Mortar Properties

In Section 2, the physical properties of CA mortar were discussed, and the relationship between the elastic modulus and the density of CA mortar was established based on the physical properties of CA mortar prepared in various studies. Therefore, controlling the elastic modulus of CA mortar can also effectively control its density. In this section, we explore the damage evolution of CA mortar under different physical properties. According to the specifications, the elastic modulus of CA mortar should be controlled within the range of 7000 MPa to 10,000 MPa, with increments of 500 MPa. Thus, the elastic modulus values considered are 7000 MPa, 7500 MPa, 8000 MPa, 8500 MPa, 9000 MPa, 9500 MPa, and 10,000 MPa. Corresponding densities are calculated using Equation (7). Additionally, the thickness of the CA mortar layer should be controlled between 30 mm and 60 mm, with increments of 10 mm, resulting in thicknesses of 30 mm, 40 mm, 50 mm, and 60 mm. Consequently, this section involves a total of 4 × 7 = 28 conditions, as detailed in Table 7. The train load is applied in the same manner as in the previous section, with ΔT = 90 °C. As analyzed in Section 3, the lifetime of CA mortar primarily depends on when the crack length reaches 100 mm, so the numerical simulation is halted once the crack length of the CA mortar layer reaches 100 mm.

**Figure 18:**
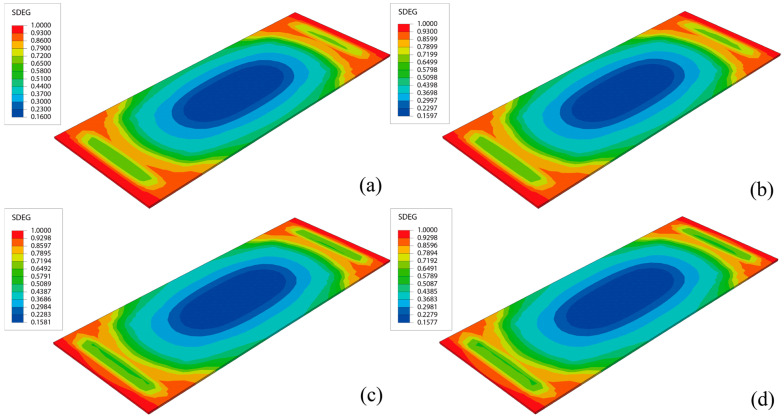
Damage distribution in CA mortar layer when crack length reaches 100 mm: (**a**) h = 30 mm, E = 7000 MPa, ρ = 1770 kg/m^3^; (**b**) h = 40 mm, E = 7000 MPa, ρ = 1770 kg/m^3^; (**c**) h = 50 mm, E = 7000 MPa, ρ = 1770 kg/m^3^; (**d**) h = 50 mm, E = 7000 MPa, ρ = 1770 kg/m^3^.

**Figure 19:**
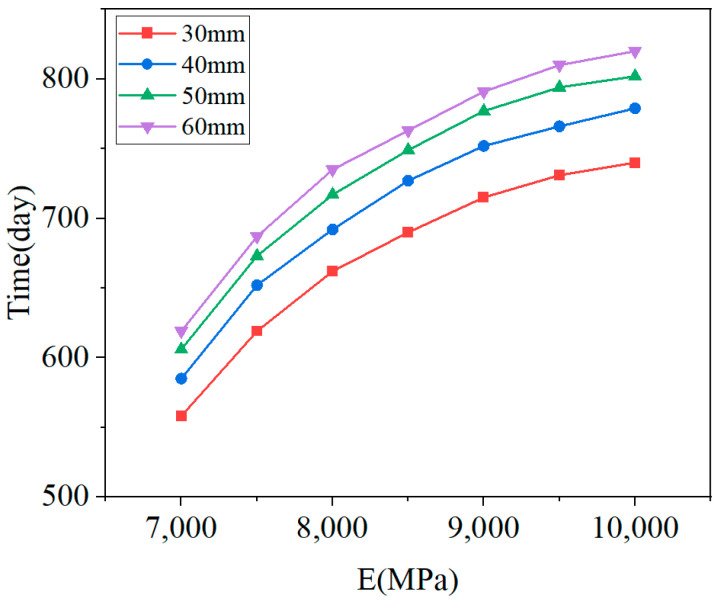
Lifetime vs. elastic modulus for different thicknesses.

**Figure 20:**
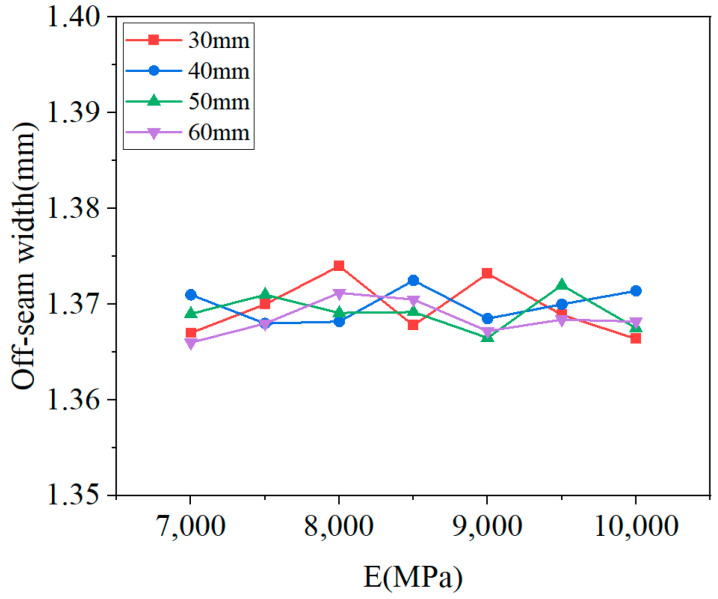
Off-seam width vs. elastic modulus for different thicknesses.

Figure 18 illustrates the damage distribution in CA mortar at the end of the calculation for four different conditions. The damage distribution at the end of the lifetime is essentially the same across all conditions, with the most severe damage consistently located at the corners of the layer. This indicates that the process of damage development is similar for all conditions. Although the damage distribution at the end of the calculation is very similar, the lifetimes under the 28 conditions vary. Figure 19 shows the relationship between lifetime and elastic modulus for different thicknesses, while Figure 20 depicts the relationship between off-seam width and elastic modulus. When the lifetime is reached, the maximum off-seam width under different conditions is almost the same, fluctuating within a fixed range and not yet reaching 2 mm. Table 4 also provides detailed lifetimes and maximum off-seam widths for different conditions. As the elastic modulus increases, the lifetime also increases, but the rate of increase in lifetime slows down as the elastic modulus becomes higher. This suggests that while increasing the elastic modulus of CA mortar can extend its service life, the cost of preparation will inevitably rise. When the elastic modulus reaches a certain level, further increases in lifetime are no longer significant, and the cost-effectiveness diminishes. For instance, with a thickness of 30 mm, the lifetime of CA mortar with an elastic modulus of 9000 MPa increased by 20.8% compared to that of 7000 MPa. However, the lifetime of CA mortar with an elastic modulus of 10,000 MPa only increased by 3.5% compared to 9000 MPa. Additionally, based on the distribution of elastic modulus values of CA mortar specimens from various experiments, as shown in Figure 3, it was observed that the elastic modulus of most CA mortar samples falls within the range of 8000 to 9000 MPa. Given this distribution, further increasing the elastic modulus beyond 9000 MPa during material preparation poses significant challenges. Therefore, it is recommended that the elastic modulus of CA mortar be set at 9000 MPa. Comparing with the distribution of the elastic modulus of CA mortar in Figure 3, it is found that most of the elastic modulus of CA mortar is in the range of 8000~9000 MPa, so it can be roughly considered that the recommended modulus of CA mortar is in the range of 8000 MPa~9000 MPa. The recommended modulus is 9000 MPa in this paper, which considers the temperature gradient loads and train loads defined by actual service frequencies; the optimization results are given closer to the engineering reality. The effect of changing the thickness of the CA mortar layer is similar to that of the elastic modulus. Although increasing the thickness can improve the lifetime of CA mortar, the increase in lifetime diminishes as the thickness becomes greater, and increasing the thickness also leads to higher costs and reduced cost-effectiveness. For example, at an elastic modulus of 7000 MPa, the lifetime of CA mortar with a thickness of 50 mm is increased by 8% compared to 30 mm, but the lifetime of CA mortar with a thickness of 60 mm only increases by 2% compared to 50 mm. What’s more, as the slab track needs to be transported to the installation site after each section has been prefabricated, reducing the thickness of the CA mortar layer can make the track delivery process easier. Thus, a thickness of 50 mm is recommended for CA mortar.

The model established has been experimentally validated using site test results. In contrast to existing studies that primarily rely on empirical tests or simplified numerical simulations, this study comprehensively accounts for time-varying temperature gradient loads and train loads defined by actual service frequencies, coupled with maintenance criteria adopted in fatigue life predictions. Consequently, the life prediction and optimization results for CA mortar exhibit enhanced fidelity to real-world operating conditions. Therefore, the predicted service life for CA mortar with diversified parameters listed in Table 7 demonstrates substantial engineering relevance, providing quantitative references for maintenance planning in high-speed railway infrastructure.

## 5. Conclusions

This paper investigates the damage evolution and predicts the lifetime of CA mortar under combined loads of train operations and temperature gradient. Unlike previous studies that relied on simplified experimental configurations or decoupled numerical analyses, the findings of this work demonstrate enhanced real-world applicability, providing actionable implications for the optimization of high-speed railway infrastructure systems. Through numerical simulation and analysis, the following conclusions can be drawn:

1. A finite element model of the CRTS II ballastless track and a high-speed railway train dynamics model was established to study the damage evolution under combined loading. To characterize the plastic properties of CA mortar in ABAQUS, the stress–strain curves obtained from tests were used to calculate these properties;
2. Maintenance criteria were employed to assess the severity of damage. When the length of the cracked area exceeds 100 mm or the off-seam width from the track plate exceeds 2 mm, the CA mortar is considered to have reached its lifetime and requires immediate repair;
3. The evolution of crack damage in the CA mortar layer was analyzed. The damage was most severe at the edges of the layer and gradually decreased towards the center. Four consecutive adjacent elements at the corners were more damaged, leading to a crack length of up to 100 mm. The crack evolution was compared under different temperature gradients, and it was observed that as ΔT increased, the damage evolution of the CA mortar tended to accelerate significantly;
4. The distribution of off-seam damage was analyzed, showing that the off-seam width was greater at the edges of the CA mortar layer than in the center, reaching a maximum at the corners. The evolution of off-seams under different temperature gradients was compared, and it was found that as ΔT increased, the number of days until the appearance of off-seam decreased and the width increased. The CA mortar began to exhibit off-seam damage on day 225 for extreme conditions (ΔT = 135 °C/m) and on day 316 for mild conditions (ΔT = 90 °C/m);
5. The rate of crack damage development in the CA mortar layer is slightly faster than that of off-seam damage. The crack area length reaches the “immediate repair” level earlier, so the lifetime of CA mortar primarily depends on when the crack length reaches 100 mm. The length of the cracked area exceeds 100 mm in 354 days under extreme situations (ΔT = 135 °C/m) while in 566 days under mild situations (ΔT = 90 °C/m). Most CA mortar layers can serve in ballastless tracks for approximately 1 to 2 years;
6. The lifetime of the CA mortar layer was compared for different physical properties. It was found that increasing the elastic modulus and thickness of the CA mortar layer is beneficial for extending its lifetime, but the cost-effectiveness significantly decreases with further increases in both properties. It is recommended that the elastic modulus and thickness of the CA mortar be set to 9000 MPa and 50 mm, respectively.

Although this study provides valuable insights, several limitations should be acknowledged. Firstly, the finite element model still incorporates simplifications of the actual track structure, such as the interaction between the CA mortar layer and the surrounding environment. Secondly, the damage constitutive model employed in this study may not fully account for the anisotropic behavior and microstructural characteristics of CA mortar. Furthermore, the validation of the simulation results was constrained by the limited availability of experimental data, especially under long-term service conditions.

In subsequent research, several promising directions can be investigated. Firstly, multi-physics coupling analyses could be conducted to investigate the combined effects of temperature, humidity, and mechanical loading on the performance of CA mortar. Secondly, multi-scale modeling approaches could be employed to explore the damage mechanisms of CA mortar, spanning from the microstructural level to the macroscopic level. Additionally, long-term field monitoring and experimental studies should be carried out to validate the lifetime prediction model and optimize maintenance strategies.

## Figures and Tables

**Table 1:** Parameters for CRTS II ballast track.

Structure	Geometric Dimensions	Material Properties
Steel rail	CHN 60 rail	*ρ* = 7850 kg/m^3^ *E* = 2.1 × 10^11^ Pa *λ* = 0.3
Fastener	Spacing 0.65 m	*α* = 1.18 × 10^−5^ m/°C *k_x_* = 50 kN/mm *k_z_* = 35 kN/mm *k_y_*: 9 kN/per group
Track plate	6450 × 2550 × 200 (mm)	C55 concrete
CA mortar layer	6450 × 2550 × 30 (mm)	*α* = 1.3 × 10^−5^ m/°C *ρ* = 1770 kg/m^3^ *E* = 7 × 10^9^ Pa *λ* = 0.35 Damage parameters: see Figure 2
Base plate	Upper side width: 2950 mm Lower side width: 3250 mm Height: 300 mm	C40 concrete
Rigidity of the roadbed	----	76 MPa/m

**Table 2:** Parameters for CR 400AF.

Structural Parameters	Value
Vehicle length	25,000 mm
Vehicle width	3360 mm
Vehicle height	4050 mm
Bogie center distance	17,800 mm
Bogie wheelbase	2500 mm
Maximum Axle Weight	17 t
wheel diameter	920 mm

**Table 3:** Comparison of numerical simulation results with test results.

Comparative Parameters	Test Result	Simulation Result
Vertical force on wheel tracks (kN)	59.6~106.1	50.8~90.5
Displacement of track plates (mm)	0.1~8.7	0.08~6.5
Vertical acceleration of the rail (g)	173~926	170~825

**Table 4:** Temperature gradient loads.

Serial Number	Maximum Positive Temperature Gradient (°C/m)	Maximum Negative Temperature Gradient (°C/m)	ΔT (°C/m)
1	90	−45	135
2	80	−40	120
3	70	−35	105
4	60	−30	90

**Table 5:** High-speed train schedule on the Guangzhou-Shenzhen railway.

Time	Departure Frequency	Time	Departure Frequency
6:00~7:00	3	16:01~17:00	9
7:01~8:00	8	17:01~18:00	6
8:01~9:00	9	18:01~19:00	7
9:01~10:00	11	19:01~20:00	5
10:01~11:00	10	20:01~21:00	8
11:01~12:00	8	21:01~22:00	6
12:01~13:00	10	22:01~23:00	4
13:01~14:00	7	23:01~0:00	2
14:01~15:00	7	0:01~6:00	0
15:01~16:00	9		

**Table 6:** The days on which damage appears and reaches the criteria and the corresponding intervals to attain the criteria. (a) cracked and (b) off-seam.

(a)			
E	Damage appears	Reaches criteria	intervals
7000 MPa	300	566	266
7500 MPa	306	619	313
8000 MPa	324	662	338
8500 MPa	375	690	315
(b)			
E	Damage appears	Reaches criteria	intervals
7000 MPa	367	787	420
7500 MPa	375	840	465
8000 MPa	390	889	499
8500 MPa	447	959	512

**Table 7:** Conditions details for different elastic moduli, densities, and thicknesses.

Serial Number	h (mm)	E (MPa)	ρ (kg/m^3^)	Lifetime	Off-Seam Width (mm)
1	30	7000	1770	566	1.367
2		7500	1820	619	1.370
3		8000	1871	662	1.374
4		8500	1922	690	1.368
5		9000	1973	715	1.373
6		9500	2024	731	1.366
7		10,000	2074	740	1.366
8	40	7000	1770	585	1.371
9		7500	1820	652	1.368
10		8000	1871	692	1.368
11		8500	1922	727	1.373
12		9000	1973	752	1.369
13		9500	2024	766	1.370
14		10,000	2074	779	1.371
15	50	7000	1770	606	1.369
16		7500	1820	673	1.371
17		8000	1871	717	1.369
18		8500	1922	749	1.369
19		9000	1973	777	1.366
20		9500	2024	794	1.372
21		10,000	2074	802	1.368
22	60	7000	1770	619	1.366
23		7500	1820	687	1.368
24		8000	1871	735	1.371
25		8500	1922	763	1.371
26		9000	1973	791	1.367
27		9500	2024	810	1.368
28		10,000	2074	820	1.368

## Data Availability

The original contributions presented in this study are included in the article. Further inquiries can be directed to the corresponding author.
